# Granule Protein Processing and Regulated Secretion in Neutrophils

**DOI:** 10.3389/fimmu.2014.00448

**Published:** 2014-09-19

**Authors:** Avinash Sheshachalam, Nutan Srivastava, Troy Mitchell, Paige Lacy, Gary Eitzen

**Affiliations:** ^1^Department of Cell Biology, University of Alberta, Edmonton, AB, Canada; ^2^Pulmonary Research Group, University of Alberta, Edmonton, AB, Canada; ^3^Department of Medicine, University of Alberta, Edmonton, AB, Canada

**Keywords:** exocytosis, Rho GTPase, protein sorting, secretion, cytokine

## Abstract

Neutrophils are part of a family of granulocytes that, together with eosinophils and basophils, play an essential role in innate immunity. Neutrophils are the most abundant circulating leukocytes and are vital for rapid immune responses, being recruited to sites of injury or infection within minutes, where they can act as specialized phagocytic cells. However, another prominent function of neutrophils is the release of pro-inflammatory compounds, including cytokines, chemokines, and digestive enzymes, which are stored in intracellular compartments and released through regulated exocytosis. Hence, an important feature that contributes to rapid immune responses is capacity of neutrophils to synthesize and store pre-formed pro-inflammatory mediators in specialized intracellular vesicles and thus no new synthesis is required. This review will focus on advancement in three topics relevant to neutrophil secretion. First, we will examine what is known about basal level pro-inflammatory mediator synthesis, trafficking, and storage in secretory compartments. Second, we will review recent advancements in the mechanisms that control vesicle mobilization and the release of pre-formed mediators. Third, we will examine the upregulation and *de novo* synthesis of pro-inflammatory mediators by neutrophils engaged at sites of infection.

## Introduction

Neutrophils are the most abundant leukocytes in blood, comprising 60–70% of all circulating white blood cells, and therefore, make up an essential part of both innate and adaptive immunity. Neutrophils are highly mobile and provide rapid responses to infection via phagocytosis of pathogens or release of potent pro-inflammatory mediators that chemically incapacitate pathogens and infected cells. Neutrophils are also recruited to the site of injury following trauma, resulting in an acute inflammatory response. Their short-life span allows for quick resolution of inflammation and can promote wound healing through stimulation of local tissue remodeling and chemoattraction of macrophage (1). Neutrophil immunity function depends on both the biogenesis of granules that store proteins with active antimicrobial activity, and on their ability to generate oxidative burst. Defects in either of these two processes results in severe immunodeficiency such as neutropenia, neutrophil-specific granule deficiencies, or chronic granulomatous disease when oxidative burst is lacking (2). In this review, we will consider the protein processing events that occur in neutrophils, resulting in their initial granulocytic morphology, and protein transitions that occur to activate neutrophil “attack” mode.

## Granulocytopoiesis

### Neutrophil differentiation from bone marrow

Mature neutrophils are differentiated from hematopoietic stem cells in the bone marrow in a process termed granulocytopoiesis or granulopoiesis (3). New neutrophils are produced daily in high numbers, up to 10^11^ in healthy individuals, which can increase several fold during infection (4). Consequently, neutrophil homeostasis is highly regulated to control their numbers. Granulocyte-colony-stimulating factor (G-CSF) is the principal cytokine regulating granulocytopoiesis. Lack of G-CSF production impairs granulocytopoiesis, resulting in neutropenia and severe immune deficiency (5, 6). Hematopoietic stem cell differentiation into granulocytes is regulated by the coordinated expression of three key myeloid transcription factors, GFI-1, PU.1, and members of the CCAAT enhancer binding protein family (C/EBPs). The balance between PU.1 and C/EBPα determines whether myeloblasts differentiate into granulocytes (high C/EBPα) or monocytes (high PU.1) (7).

The chemokines CXCL12 and CXCL2 and their neutrophil receptors, CXCR4 and CXCR2, respectively, regulate the release of neutrophils from the bone marrow. CXCR4–CXCL12 signaling promotes bone marrow retention, while CXCL2–CXCR2 promotes their release in what has been described as a tug of war model. The contribution of these chemokine signaling pathways to the regulation of neutrophil trafficking from bone marrow has been recently reviewed (8).

### Neutrophil differentiation from stem cells

Neutrophils have been developed from human embryonic stem cells (hESCs) in culture. High yields were achieved by growing hESC in co-culture with semi-confluent OP9 stromal cells to stimulate hESC differentiation (9). The OP9 stromal cell line, which is derived from the osteopetrotic mouse, was specifically selected since they lack production of macrophage-colony stimulating factor (M-CSF) and thus support neutrophil rather than macrophage differentiation (10, 11). The procedure was further improved using a unique cytokine cocktail to yield highly functional neutrophils capable of chemotaxis, phagocytosis, oxidative burst, and bacterial killing (12, 13). Recently, protocols have been reported that differentiated neutrophils can be prepared from induced pluripotent stem cells (iPSCs) (14–16). These are significant advancements since the ability to obtain functional neutrophils from hESCs, iPSCs, and even patient-derived iPSCs may progress toward their eventual use in the treatment of hematopoietic disorders (17, 18).

## Neutrophil Granularity

### Sequential synthesis model

**Figure 1:**
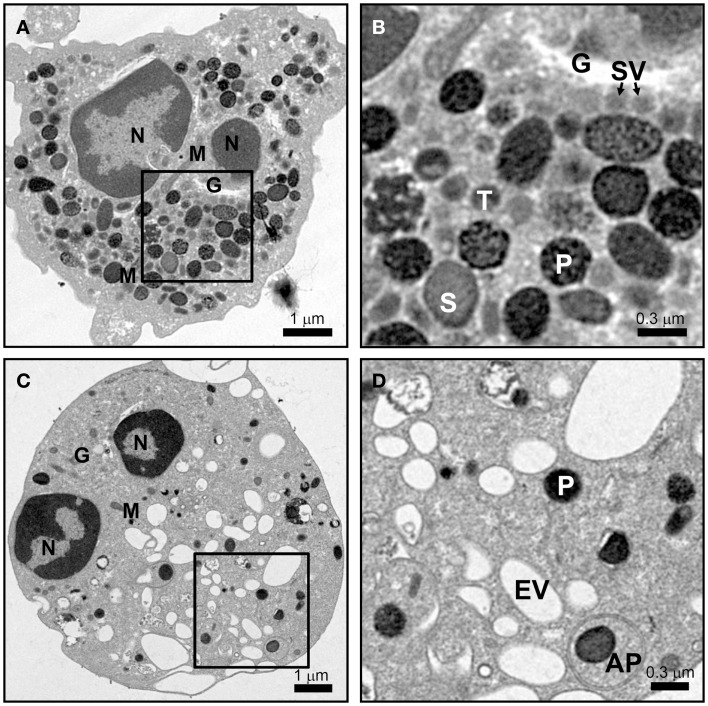
**Neutrophil morphology as observed by electron microscopy**. Neutrophils were isolated from human peripheral blood, and processed for electron microscopy after incubation for 15 min at 37°C with vehicle **(A,B)** or 10 μM cytochalasin B and 5 μM fMLF **(C,D)** as described in Ref. (22). **(A,B)** The cytosol of a resting cell is filled with vesicles, with primary granules (P) staining intensely dark with DAB, while secondary (S) and tertiary (T) granules show more translucent staining. Secretory vesicles (SV) are in close proximity to the Golgi complex (G). Few mitochondria (M) are observed. **(C,D)** After stimulation only a few dense primary granules (P) remain and many empty vesicles (EV) appear. A double membrane autophagosome (AP) was observed to form around a primary granule; however, the relevance of this observation is unknown. Scale **(A,C)** = 8 μm × 8 μm, 9,100× magnification; **(B,D)** = 2.4 μm × 2.4 μm, 27,600× magnification.

Mature neutrophils emerge in the blood devoid of any proliferative capacity, but fully capable of launching an immune response. Ultrastructural images of mature neutrophils reveal a characteristic multi-lobed nucleus, very few mitochondria, a small Golgi structure, and a highly granular cytosol that is packed with vesicles (Figure 1A). The presence of different granule subsets is revealed by their varying size and intensity of stained with the peroxidase-reactive dye, 3,3′-diaminobenzidine (Figure 1B). Granule subsets are also distinguished by their protein content and their propensity for mobilization, which will be further discussed in following sections (19–21).

**Figure 2:**
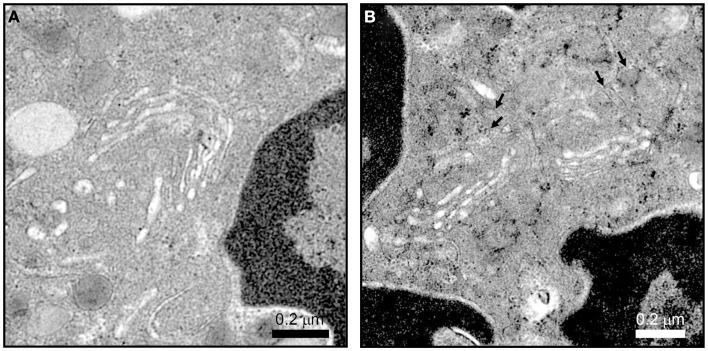
**Neutrophil Golgi complex as observed by electron microscopy**. Neutrophils processed for electron microscopy as described in Ref. (22) show a small stacked Golgi with an increase in electron dense vesicle (*arrows*) after stimulation. Conditions were incubation with vehicle **(A)** or 10 μM cytochalasin B and 5 μM fMLF **(B)** for 15 min at 37°C. Scale, 2.4 μm × 2.4 μm, 27,600×magnification.

The difference in protein contents among neutrophil granule subsets is not driven by protein sorting. Instead, different granules are sequentially formed during neutrophil differentiation in what has been described as a targeting by timing model (23). According to this model, as different granule proteins are synthesized during different stages of neutrophil differentiation several granule subsets are generated (24–26). Neutrophils have at least three distinct granule subsets: (i) primary or azurophilic granules, which contain potent hydrolytic enzymes (e.g., elastase) and myeloperoxidases (MPO), (ii) secondary or specific granules, which contain high levels of the iron-binding protein lactoferrin, and (iii) tertiary or gelatinase granules, which contain matrix metalloproteinases (Figure 1B). Secondary and tertiary granules contain an overlapping set of proteins; however, all granules have distinctive buoyant densities and can be isolated by density gradient centrifugation (27). Recently, a fourth granule population has been described that was enriched in the microbial lectin ficolin-1. Ficolin-1 is found in tertiary granules; however, the authors found a second pool of ficolin-1-rich/gelatinase-poor granules with an elevated exocytosis propensity (21, 28). The importance of these granules may be to provide rapid release of pattern recognition molecules to activate the lectin complement pathway (28). Secretory vesicles (SV) also appear in density gradients that contain albumin indicative of their formation via endocytosis. SV can also be formed in mature neutrophils from a small Golgi structure (Figures 2A,B). These contain cytokines synthesized during immune activation indicative of an active biosynthetic pathway (29, 30), which will be discussed further in Section “Neutrophil *De Novo* Synthesis of Pro-Inflammatory Mediators.”

### Granule protein sorting

Neutrophil granule proteins undergo a series of co- and post-translational processing and targeting events during sorting to their granule sub-compartment. Most, if not all, proteins packaged into neutrophil granules enter through the biosynthetic process as deduced by the presence of amino-terminal signal sequences for co-translational translocation into the endoplasmic reticulum (ER). The emergence of this “pre” sequence from the ribosome associates with the signal recognition particle, which drives co-translation into the lumen of the ER, followed by post-translational processing as proteins traverse the Golgi complex (e.g., protein maturation via glycosylation).

**Figure 3:**
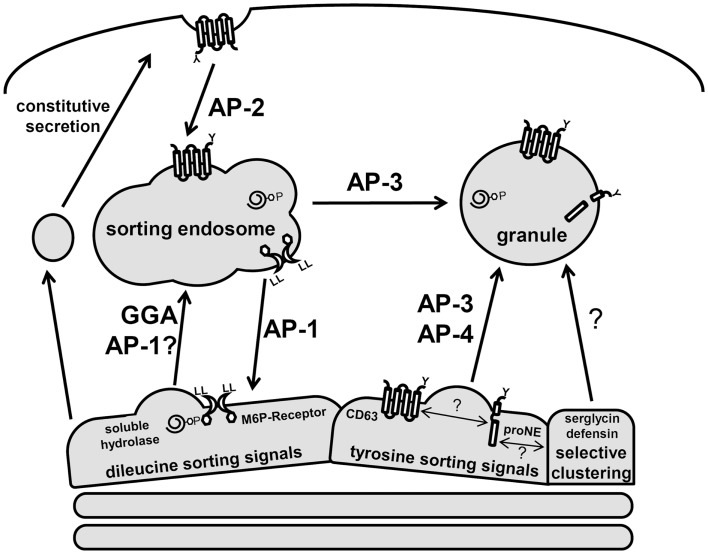
**Schematic of neutrophil protein sorting pathways**. Three prominent trafficking pathways for granule proteins are depicted: AP-1/GGA-dileucine based sorting, AP-3/AP-4-tyrosine-based sorting, and selective clustering-based sorting. Soluble hydrolases contain M6P signals and are sorted via the M6P-receptors, which contain dileucine signals. These complexes are shuttle to the sorting endosome through GGA and AP-1-dependent pathways. In the endosome, a drop in pH results in dissociation of the receptor–cargo complex with the retrieval of the M6P-receptor by an AP-1 dependent pathway. Serine proteases (neutrophil elastase, proNE) and *trans*-membrane proteins (CD63) contain tyrosine signals and are sorted directly to granules via AP-3/AP-4. Many granule proteins contain neither dileucine nor tyrosine signals and are thought to sort to granules via a clustering-based pathway since this occurs for many granule proteins that are highly concentrated. CD63 trafficking is unique as it can be found on several neutrophil membranes and may act to selectively retrieve granule proteins that escape normal trafficking via constitutive secretion.

The precise mechanism by which proteins are targeted to neutrophil granules from the *trans*-Golgi network (TGN) is largely unknown, hampered by the fact that these cells are end-differentiated and do not synthesize granule proteins *de novo*. However, much information has been gained from analogous studies in other immune cells and the characterization of neutrophils from immunological disorders associated with sorting defects (31, 32). These studies confirm that neutrophils generate lysosome-like secretory granules both directly from the TGN and indirectly via a sorting endosomes (Figure 3). Post-TGN sorting is mediated by the multi-subunit adaptor protein complexes (APs) and the monomeric Golgi-localized γ-adaptin ear homology ARF binding (GGAs) protein. Both nucleate clathrin-coated vesicles and recruit luminal cargo for packaging into the vesicles (33). Three of the five known AP complexes function at the TGN and are likely involved in granule sorting since they have prominent roles in lysosomal sorting in other cell types (33, 34). AP-1 and GGAs direct the bulk sorting program from the TGN to sorting endosomes, while AP-3 and AP-4 shuttle cargo to granules from the sorting endosomes or directly from the TGN, bypassing the sorting endosomes (35). This distinction in pathways serves an important mechanistic role since soluble cargo utilizes receptors that require a dissociation step catalyzed in endosomes. This allows the receptor to be sorted back to the TGN while the cargo is transported into granules (Figure 3).

The sorting of granule membrane proteins, including cargo receptors, depends on dileucine or tyrosine-based sorting signals present in carboxy-terminal cytosolic domains (35). The consensus dileucine sequences, DXXLL or [DE]XXXL[LI], and tyrosine sequence, [G]YXXØ (where X represents hydrophobic residues and Ø a bulky hydrophobic amino acid), interact with GGAs/AP-1 and AP-3/AP-4 complexes, respectively.

The majority of soluble hydrolases and granzymes are modified with mannose-6-phosphate (M6P) in the Golgi, which is recognized by M6P-receptors to facilitate their exit from the TGN (33). Unlike hydrolases and granzymes, many soluble granule proteases are sorted via M6P-independent mechanisms (31, 36). Targeting of the major serine protease, neutrophil elastase, as well as other proteases requires AP-3 (37). Neutrophil elastase contains a tyrosine on the cytosolic face of it *trans*-membrane pro-domain, which interacts with AP-3 presumably for its sorting (38). However, neutrophil elastase mutants that lack a pro-domain still get sorted to granules via a CD63-dependent mechanism (39). CD63, an abundant membrane protein of primary granules, is sorted by AP-3, and has been shown to play a role in granule protein quality control since it can be trafficked from many membranes to granules (40, 41). A recent study showed that CD63 and neutrophil elastase are present in a complex, highly indicative of a role for CD63 in the targeting of elastase to primary granules (42). Other serine proteases affected by CD63 granule sorting include proteinase-3 (39), and possibly the cathepsin family of proteases, which exhibit varying degrees of M6P-dependent/independent sorting (36, 43–45).

Neutrophils, as well as other granulocytes, may have acquired additional unique granule sorting mechanisms based on the fact that proteins in some granules can be tightly packed to the point of having a crystalline core. A selective aggregation of proteins destined for storage in granules would eliminate the need for distinct sorting motifs on each granule protein (24). Granule proteins, which are often cationic, may be clustered with serglycin, a major anionic proteoglycan of hematopoietic cells. Serglycin has been proposed to play a role in sorting and packing of several granule proteins including α-defensin and elastase (46, 47). Neutrophil elastase is absent from mature neutrophils in serglycin knock-out mice (48). Whether serglycin-induced clustering of granule proteins promotes granule localization via sorting or their retention in granules is still under investigation (49).

Most granule enzymes are synthesized as zymogens and undergo a proteolytic processing step that depends on the acidic pH levels within this lysosome-like compartment. The proteolytic cleavage of granule pro-domains converts these enzymes into their active forms and thus many granule proteins are maintained inactive until the proper compartment is reached. Some pro-domains have also been shown to have a role in sorting. The amino-terminal pro-peptide of pro-MPO has been shown to facilitate its targeting to primary granules (50). Unlike MPO, serine proteases (e.g., elastase and cathepsin G) are synthesized with both amino-terminal and carboxy-terminal pro-peptides that do not seem to be involved in their sorting (51).

### Immune disorders from granule protein sorting defects

Granule protein trafficking disorders often manifest as lysosomal storage disorders (LSDs), which are a large group of metabolic diseases that result from deficiencies in specific lysosomal enzymes or defects in lysosome biogenesis (31, 52). For instance, defects in the ability to generate M6P sorting signals in the Golgi result in the LSDs mucolipidosis II (also referred to as I-cell disease) and mucolipidosis III. Mutations in GlcNAc-phosphotransferase are believed to be the primary genetic defect in mucolipidosis. GlcNAc-phosphotransferase activity is absent in MLII and altered in MLIII (53). Studies of patients with mucolipidosis revealed neutropenia, with a major reduction in the number of neutrophils in MLII, but less severe in MLIII patients (53, 54). There have been no reports of neutrophil function studies from these patients, but presumably granules would lack most soluble hydrolases since these are sorted by the M6P pathway. However, studies of B lymphocytes showed that a portion of lysosomal enzymes were retained in granules with an unknown a back-up sorting event facilitating this partial sorting (36). The recent development of a mucolipidosis II mouse model might advance neutrophil functional characterizations (55, 56). These studies could reveal the prominence of M6P-independent sorting pathways in neutrophils and whether these can facilitate the partial retention of enzymes normally sorted by M6P.

Hermansky–Pudlak syndrome type 2 is also a LSD disorder that results from a mutation in AP3B1, which encodes a subunit of the AP-3 complex. The type 2 form includes immunodeficiency associated with neutropenia and partial albinism. The basis of this disease was first discovered as a similar autosomal recessive disease of dogs, canine cyclic hematopoiesis (38). The lack of AP-3 sorting resulted in reduced levels of neutrophil elastase and gelatinase in granules, but normal levels of other enzymes such as MPO and proteinase-3, which reside in the same granule fraction as elastase (57). This reveals the divergence in sorting pathways in neutrophils; AP-3 for neutrophil elastase versus GGA/AP-1 for MPO. Interestingly, AP-3 deficiency leads to congenital neutropenia in humans and cyclic neutropenia in dogs (38, 58), even though both are linked to the lack of neutrophil elastase sorting. Neutrophil elastase mutations cause a much more severe congenital neutropenia phenotype in humans (59). It has been suggested that increased apoptosis of myeloid precursors in patients carrying mutations in the elastase gene could lead to a maturation arrest of myelopoiesis and this triggers the more severe neutropenia phenotype (60).

Although neutrophil elastase is a soluble protein, there is evidence that in an intermediate stage of its processing it is a disulfide bonded *trans*-membrane protein (38). Upon enzymatic removal of the carboxy-terminal pro-domain, the protein maintains a *trans*-membrane conformation capable of associating with AP-3 complexes. Membrane-associated neutrophil elastase utilizes a tyrosine-based sorting signal that is located in the carboxy-terminal domain of the protein. Most elastase mutations associated with severe congenital neutropenia result in the removal of this sorting signal (38). Horwitz et al. have proposed that the localization of the enzyme in the lumen of granules or on the plasma membrane may regulate the differentiation pattern of myeloid progenitor cells into monocytes or granulocytes. This suggests that neutropenia may result from a defective differentiation switch between the monocytic and granulocytic lineage of myelopoiesis (61, 62). This is an appealing hypothesis that may account for the observation that granulocytes and monocytes reciprocally cycle in numbers during normal hematopoiesis and for the typical increase of monocyte blood counts observed in the majority of severe congenital neutropenia patients. Hence, not only granule sorting pathways are important for granule biogenesis but also in determining the population of cell types during hematopoiesis.

## Protein Processing in Activated Neutrophils

Three functions that involve protein processing occur in neutrophils when activated for an immune response: (1) neutrophil migration up chemotactic gradients to sites of infection, (2) destruction of pathogens or injured/infected cells through oxidative burst and phagocytosis, and (3) mobilization of granules and release of inflammatory mediators. In this section, we review these functions with a particular focus on the mobilization of granules and the release of pre-formed inflammatory mediators.

### Neutrophil chemotaxis

Upon activation, neutrophils migrate out of blood vessels following gradients of chemoattractants in a process known as chemotaxis. Neutrophils respond to a wide range of “neutrophil-active” chemoattractants, including chemokines and cytokines (CXCL8, IFNγ), complement (C5a), eicosanoids (LTB_4_), and pathogen-derived peptides such as formylated met–leu–phe (fMLF) (63, 64). Chemotaxis begins with neutrophil recruitment from circulation through physical interactions of neutrophil-specific adhesion molecules. The neutrophil adhesion cascade is the focus of recent reviews and therefore only briefly described here (65, 66). Neutrophils are initially tethered by selectin interactions, which can be upregulated on the surface of endothelial cells (E-selectin) and leukocytes (L-selectin). The lectin-like domains of selectins interact with sialylated carbohydrate groups present on surface proteins. This results in the characteristic rolling of neutrophils along the luminal side of the endothelium. Reduced velocity allows neutrophils to recognize chemokines on the surface of the endothelium, which activates integrins. Neutrophils will be released from low-affinity selectin interactions unless firm adhesive contacts are made between integrins and endothelial intracellular adhesion molecules (ICAMs). Integrin ligation triggers intracellular signaling for cytoskeletal rearrangement, which polarizes neutrophils and drives transmigration through the endothelium (diapedesis). Integrin signaling is essential as deficiencies result in leukocyte adhesion deficiency, which not only causes defects in chemotaxis and adhesion but also in phagocytosis and respiratory burst (2).

Regulation of the adhesion and chemotaxis requires the coordination of multiple protein signaling and processing events, which result in cytoskeletal rearrangements that generate cell polarity (67). The activation of distinct Rho proteins at the front (Rac1) and back (RhoA) of neutrophils create actin formations that drive movement (68). Rho proteins are central regulators of multiple intracellular processes, and it is interesting how signaling downstream from Rho has evolved to coordinate cytoskeletal remodeling in conjunction with other processes required for immune cell functions. These include many non-cytoskeletal-related functions such as regulation of gene transcription (69–72), calcium flux (73, 74), and oxidative burst (75–77).

Chemokines interact with G protein-coupled receptors (GPCRs) on the surface of neutrophils. One pathway activated by GPCRs is the lipid kinase pathway. PI3-kinase is activated, which converts phosphatidylinositol-4,5-bisphosphate [PI(4,5)P_2_] into phosphatidylinositol-3,4,5-triphosphate [PI(3,4,5)P_3_] at the plasma membrane. PI(3,4,5)P_3_ stimulates F-actin reorganization at the leading edge driving lamellipodia formation. The Rac GEFs, Vav1, and P-Rex1, bind PI(3,4,5)P_3_ via pleckstrin homology domains, activating Rac, which leads to the production of branched actin filaments at the leading edge (78–80). High levels of the phosphatase PTEN are present in the trailing edge of neutrophils, which consumes PI(3,4,5)P_3_. Thus, a gradient of PI(3,4,5)P_3_ is established toward the leading edge, which promotes polarization and directional motility (81). A recent report examined neutrophils derived from a conditional PTEN knock-out and showed it not only functions in polarization but also prioritizes chemoattractant signals. Normal neutrophils were shown to prefer bacterial chemoattractants over endogenous chemokines, but this preferential selection was lost in the PTEN knock-out (82).

Opposing roles for the two Rho proteins, RhoA and Cdc42, in the trailing edge (or uropod) were shown by examining neutrophils devoid of these proteins. In the absence of RhoA, neutrophil priming was unregulated, resulting in hyper-responsive activation and uncoordinated motility (83). Cdc42, on the other hand, was required specifically to maintain neutrophil polarity (84). WASp, a downstream Cdc42 effector, regulated polarity through the CD11b integrin, which recruits the microtubule end binding protein, EB1, to capture and stabilize microtubules at the uropod. This would allow the generation of “pushing” forces from the back of neutrophils. Cross-talk between Rho proteins has been an area of intense investigation (68) and these results show how Rho/Cdc42 signaling cross-talking can coordinately control neutrophil chemotaxis while at the same time minimize other immunity functions such as degranulation.

### Phagocytosis and respiratory burst

Neutrophil phagocytosis has been the focus of several reviews (85–87). For the purpose of this review, we will limit our consideration to the protein processes involved in NADPH oxidase assembly and how this is directed primarily to the phagosomal membrane.

The rapid immunological response of neutrophils not only depends partly on pre-formed granule proteins with antimicrobial activity but also on the ability to generate respiratory burst activity. Deficiency in respiratory burst results in a severe immunodeficiency called chronic granulomatous disease (2). Chronic granulomatous disease is linked to genetic abnormalities in any one of the five “phox” proteins, which comprise the NADPH oxidase complex. The small GTPase Rac is a sixth component that is physically part of the oxidase complex that regulates its function (75).

The NADPH oxidase complex generates reactive oxygen species (ROS) that are crucial for antimicrobial activity and antigen processing. However, excessive ROS production causes tissue damage and oxidative stress and therefore neutrophils must precisely control both the location and timing of NADPH oxidase activity. In unstimulated neutrophils, three oxidase subunits, p47-phox, p67-phox, and p40-phox, are cytosolic. Rac2 is also cytosolic, bound to its natural inhibitor RhoGDI. The remaining two subunits, gp91-phox and p22-phox, form the membrane-spanning catalytic core of cytochrome b558. Formation of the holoenzyme allows electrons to follow from NADPH bound at the cytosolic face of the complex, to FAD-bound cytochrome b558, which has two heme-bound oxygen groups that accept the electrons and produce superoxide.

The oxidase complex can be preferentially incorporated into the phagosome during pathogen endocytosis, which minimizes collateral tissue damage. However, residual oxidase complex at the plasma membrane or its activation in the absence of phagocytosis results in the release of ROS extracellularly. Three regulatory steps may facilitate the phagosomal-predominant spatial activation of the oxidase complex. p47-phox phosphorylation leads to a conformational change that exposes two amino-terminal SH3 domains, which interact with the proline-rich region of the membrane-bound p22-phox (88). Assembly that occurs on the plasma membrane is susceptible to in rapid dephosphorylation, resulting in unproductive oxidase assembly. Second is the regulation of the p40-phox subunit by PI3-kinase. This effect is mediated by the PX domain of p40-phox binding to PI(3)P, which results in an open conformation allowing NADPH access to the catalytic core (89, 90). PI(3)P is enriched on phagosome membranes, which facilitates the spatial restriction of oxidase activity to the phagosome rather than at the plasma membrane where the predominant phosphoinositol lipids are PI(4,5)P_2_ and PI(3,4,5)P_3_. A third regulatory step involves Rac. Rac is activated by guanine nucleotide exchange factors (GEFs) from the Vav or P-Rex families (78–80, 91). The function of these GEFs depend on membrane recruitment via increased levels of PI(3,4,5)P_3_ (92). However, this create a spatial paradox since PI(3,4,5)P_3_ is localized to the plasma membrane and not phagosomes. This is resolved by the oxidase complex showing preference for assembly with Rac2 over Rac1 (91, 93, 94). While Rac1 is predominantly localized to the plasma membrane, Rac2 has less affinity for the plasma membrane, likely due to fewer polybasic residues in its carboxy-terminus (95). This may result in spontaneous disassembly from the oxidase complex in the presence of high levels of PI(4,5)P_2_ and PI(3,4,5)P_3_, unless rapidly endocytosis occurs and the highly acidic phosphoinositides are metabolized.

Interestingly, the Rac GEF Vav has also been shown to affect oxidase assembly through a secondary affect involving the p40-phox subunit (96). This may involve other downstream effectors of Rac, which includes the serine kinase PAK1, which has been shown to phosphorylation p47-phox (97). In addition, RhoA has recently been shown to downregulate ROS production (98). The RhoA signaling pathway was linked to cytoskeletal remodeling, which would provide for an elegant way to downregulate oxidase activity during chemotaxis. Further studies of the convergence of multiple signaling pathways will be needed to reveal the full control of oxidase activity.

### Granule mobilization and regulation of exocytosis

**Figure 4:**
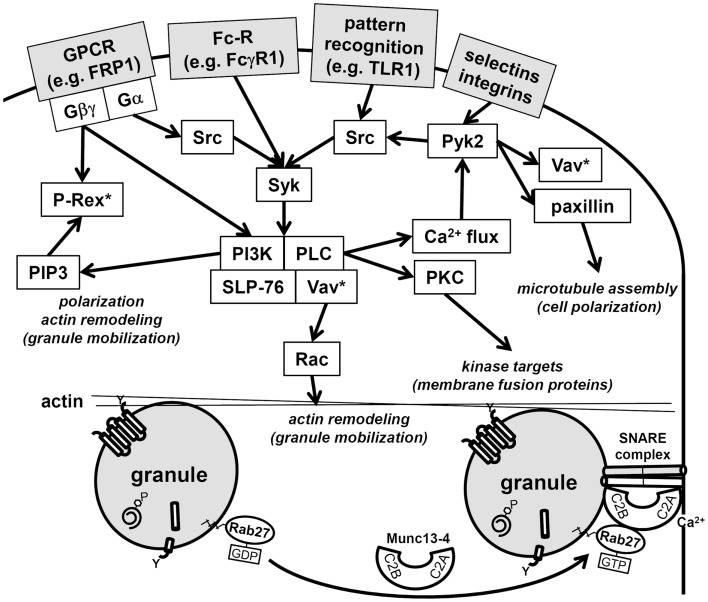
**Schematic of neutrophil signaling pathways regulating degranulation**. Two pathways that regulate granule mobilization are depicted: upstream kinase cascade and downstream fusion machinery. Activation of neutrophils through surface receptors triggers the activation of a kinase cascade. Central downstream effectors of these kinases target cytoskeletal remodeling, these include Vav, which activates Rac, paxillin, which facilitates microtubule polarization, and the generation of PI(3,4,5)P3, which facilitates polarization and actin remodeling. Note that Rac is activated at multiple points in the signaling pathways (Vav*, P-Rex*) and hence may be needed for several immune cell functions in addition to degranulation. Granule tethering is regulated by Rab27 recruitment of Munc13-4. Munc13-4 is a calcium-sensitive link between Rab function and the fusion machinery of SNAREs. The Munc13-4:SNARE interaction requires calcium flux and is targeted by PKC, which are activated in the upstream kinase cascade.

Neutrophils, upon stimulation undergo a series of immediate changes without the need for *de novo* synthesis of proteins (compare Figures 1A and 1B vs. 1C and 1D). Exocytosis, also known as degranulation in neutrophil, is the release pre-formed mediators from granules. Granule subsets are markedly different in their capacity for mobilization in response to stimulation (99, 100). Granules formed during the later stages of granulocytopoiesis are more prone to undergo exocytosis than granules formed during the earlier stages. A recent study reported exocytosis levels of 100% for SV, 38% for tertiary granules, 22% for secondary granules, and only 7% for primary granules after stimulation (28). The specific steps of exocytosis involve granule translocation toward a target membrane via actin remodeling and microtubule assembly, followed by tethering and docking through the sequential action of the core fusion machinery of Rab and SNARE proteins (Figure 4) (101, 102).

Neutrophil granule contents, which include MPO, elastase, lactoferrin, and matrix metalloproteinases, possess potent antimicrobial activity but are also highly cytotoxic. Therefore, their release is highly regulated by binary signals to minimize aberrant degranulation. The first of these binary signals is adhesion-dependent while the second involves activation of immune receptors by ligand interactions. The adhesion-dependent step *in vivo* involves β2-integrins (100), which can be reconstituted *in vitro* by adhesion to biological surfaces or the addition of actin depolymerizing agents (22, 103, 104). The adhesion-dependent signaling cascade for degranulation operates through the Src kinases Fgr and Hck. Neutrophils from double knock-out *hck*^−/−^ *fgr*^−/−^, while still able to adhere to substrate, fail to undergo degranulation in response to tumor necrosis factor (TNF) (105). However, Src kinases are well known to be membrane proximal regulators of neutrophil degranulation during receptor-mediated activation, which comprises the second half of the binary signal (106, 107). For example, degranulation requires immune receptor–ligand interactions such as formylated peptides binding to GPCRs. This also triggers the activation of Src kinases, and hence it is curious why binary signals are needed that seemingly activate the same signaling cascade. Binary signals may be needed to reach an activation threshold for degranulation or perhaps distinct downstream pathways are activated. One downstream pathway specifically activated by the receptor-mediated kinase cascade is Ca^2+^ release from intracellular stores (107). Increasing concentrations of Ca^2+^ are responsible for the hierarchical release of neutrophil granules in the order of secretory vesicles > tertiary granules > secondary granules > primary granules (99).

Cross-talk between the binary degranulation signals makes it difficult to clearly define each signaling pathways. For example, recently a role for the highly abundant non-receptor tyrosine kinase, proline-rich kinase 2 (Pyk2), was described for degranulation (108). Pyk2 undergoes auto-phosphorylation after integrin ligation in calcium-dependent manner, allowing its association with the Src-family kinases. Furthermore, studies with *pyk2*^−/−^ cells have shown its effect is mediated through paxillin and Vav, which are both phosphorylated by Pyk2 (108). Vav is a direct activator of Rac, which remodels actin to facilitate degranulation (78), while paxillin serves as an important scaffolding protein and has a direct effect on microtubule assembly, which may contribute to polarized granule motility (109). Thus, Pyk2 signaling may be central to the transduction of multiple upstream signals into downstream granule mobilization and degranulation (Figure 4).

Downstream events are regulated by distinct core fusion machinery that drives granule docking and exocytosis at specific target membranes. Initially, Rab proteins tether vesicles to target membranes, then SNARE proteins catalyze fusion (102). Neutrophil granules fuse with target membranes when v-SNARES pair with their corresponding t-SNARES. VAMP-2 is predominantly localized to SV and tertiary granules, whereas VAMP-7 is predominantly localized to primary granules (110, 111). It seems that the membrane density of VAMP-2 provides a functional role in mobilization, docking, and fusion to the plasma membrane, while VAMP-7 redirects vesicles to fuse predominantly with phagosomes (110, 112).

Numerous additional regulatory proteins interact with the core fusion machinery of Rabs and SNAREs. Among these, the Munc family of proteins is important regulators of SNARE complex formation (101). Munc13-4 (also known as UNC13D) is highly expressed in neutrophils and interacts with Rab27, which is the Rab that direct granules to dock at the plasma membrane. Munc13-4 tethers granules to the plasma membrane for exocytosis via two calcium-sensitive lipid bind C2 domains (Figure 4) (113). Neutrophil subfractionation revealed that high levels of Munc13-4 rapidly translocate from the cytosol to granules upon stimulation with a secretagogue like fMLF (114). In this study, Munc13-4 was shown to associate with secondary and tertiary granules and down-regulation of Munc13-4 using small interfering RNA decreased their exocytosis (114). Recently, STK24 and CCM-3, two regulators of neutrophil exocytosis, were shown to interact with Munc13-4 (115). Lack of either STK24 or CCM-3 inhibited degranulation; however, they were shown to play opposing roles in the regulation of Munc13-4. Whereas STK24 inhibited Munc13-4 function by binding its C2B domain, CCM-3 counteracted STK24-mediated C2B inhibition (115). The STK24/CCM-3 complex seems provide an important additional control mechanism to halt aberrant degranulation.

## Neutrophil *De Novo* Synthesis of Pro-Inflammatory Mediators

Neutrophils are well characterized for their ability to synthesize and secrete over 70 different cytokines, chemokines, and growth factors. Although several of these have been characterized at the mRNA level only, 11 of these show controversial data for human neutrophils (116). The trafficking pathways that govern the synthesis, storage, and release of these factors are poorly understood. Several studies using immunogold staining analysis of transmission electron microscopy have revealed that neutrophils store pre-formed cytokines in secretory granules. Transforming growth factor-α (TGFα) is stored as a pre-formed mediator in secretory granules that are peroxidase-negative, suggesting localization to secondary or tertiary granules (117). Similarly, TNF-α was found as a pre-formed mediator in cytoplasmic vesicles following immunogold staining (118). However, the characteristics of these cytoplasmic vesicles were not further elucidated in this study. Other studies investigating cytokine and chemokine expression in neutrophils have indicated that mature peripheral blood neutrophils possess pre-formed IL-6, IL-12, and CXCL2 in their SV or tertiary granules (119).

In neutrophils, CD63 and CD68 are abundant membrane-bound proteins in primary and secondary granules, and both of these possess the YXXΦ motif, which interacts with AP-3/AP-4 complexes. AP-3 traffics cargo from tubular endosomes (recycling endosomes) to late endosomes, lysosomes, and related organelles, while AP-4 traffics protein from the TGN to endosomes or directly to lysosomes (33, 35). This indicates that CD63 and CD68 may be engaged in trafficking to primary and secondary granules through recycling endosomes and late endosomes. Conversely, cytokines that are secreted by neutrophils, including IL-1α/β, IL-6, CXCL8, IL-12, TNF, IFNγ, CXCL2, TGFβ, MIP-1α/β, and VEGF do not possess adaptor motifs, suggesting that they do not directly bind to adaptors in order to enter sorting pathways for their trafficking. Based on the absence of adaptor motifs in cytokines, it is likely that cytokine trafficking is mainly determined by adaptor motifs present in membrane proteins such as CD63 and CD68, which direct cytokine cargo to appropriate granular and vesicular compartments in cells.

Lipopolysaccharide (LPS) is major TLR agonist that is shed from the outer membranes of Gram-negative bacteria, which cause a significant burden of disease by inducing respiratory infections, gastrointestinal disorders, sepsis, pneumonia, and many other transmissible infections. LPS stimulation results in substantial production of TNF, a potent pro-inflammatory cytokine with cytotoxic and pyrogenic effects. Neutrophils stimulated by LPS have the ability to release TNF, potentially through granule stores (118, 120). However, the mechanisms used to secrete cytokines and chemokines has not been thoroughly investigated in neutrophils although it is evident that SNAREs and other trafficking machinery are required for exocytosis of cytokine and chemokine-carrying granules (110, 111, 121, 122).

## Conclusion

Granule exocytosis and release of their cytotoxic contents at the plasma membrane is most often an undesirable effect of inflammation. We have highlighted many of the protein processes involved in pro-inflammatory mediator synthesis and granule biogenesis. Neutrophils possess several mechanisms that promote fusion of granules with phagosomes to minimize the effect of neutrophil-induced co-lateral tissue damage. However, in many inflammatory disorders, such as acute lung injury, ischemia/reperfusion injury, severe asphyxic asthma, rheumatoid arthritis, and septic shock, excessive neutrophil degranulation is a common feature. Insight into neutrophil protein processes holds much promise in the future treatment of these diseases.

## Conflict of Interest Statement

The authors declare that the research was conducted in the absence of any commercial or financial relationships that could be construed as a potential conflict of interest.
